# A Virtual Reality-Cycling Training System for Lower Limb Balance Improvement

**DOI:** 10.1155/2016/9276508

**Published:** 2016-03-06

**Authors:** Chieh Yin, Ya-Hsin Hsueh, Chun-Yu Yeh, Hsin-Chang Lo, Yi-Ting Lan

**Affiliations:** ^1^Department of Electronic Engineering, National Yunlin University of Science and Technology, Yunlin 64002, Taiwan; ^2^School of Physical Therapy, Chung Shan Medical University, Taichung, Taiwan; ^3^Department of Product Design, Ming Chuan University, Taoyuan, Taiwan; ^4^Room of Physical Therapy, Chung Shan Medical University Hospital, Taichung, Taiwan

## Abstract

Stroke survivors might lose their walking and balancing abilities, but many studies pointed out that cycling is an effective means for lower limb rehabilitation. However, during cycle training, the unaffected limb tends to compensate for the affected one, which resulted in suboptimal rehabilitation. To address this issue, we present a Virtual Reality-Cycling Training System (VRCTS), which senses the cycling force and speed in real-time, analyzes the acquired data to produce feedback to patients with a controllable VR car in a VR rehabilitation program, and thus specifically trains the affected side. The aim of the study was to verify the functionality of the VRCTS and to verify the results from the ten stroke patients participants and to compare the result of Asymmetry Ratio Index (ARI) between the experimental group and the control group, after their training, by using the bilateral pedal force and force plate to determine any training effect. The results showed that after the VRCTS training in bilateral pedal force it had improved by 0.22 (*p* = 0.046) and in force plate the stand balance has also improved by 0.29 (*p* = 0.031); thus both methods show the significant difference.

## 1. Introduction

Advancements in medical technology have improved the survival rate of stroke patients. However, stroke survivors may have many complications after they survived, such as abnormal muscle tone and hemiparesis [1, 2]. The bilateral sides of stroke patients are often asymmetric or imbalanced, which limit their balance ability and hinder them from coordinating both sides of their bodies during movements [3]. The lack of basic functions compels stoke patients to heavily rely on external assistance for their daily activities. An important rehabilitation goal is to help stroke patients to train the hemiparesis side of their bodies, and to enhance their motor control and coordination, which eventually improves the independence ability of patients.

One of the most important objectives of stroke rehabilitation is to restore a participant's walking ability [4–7]. Walking for these patients, many times, requires a significant increase in strength and coordination. Many studies suggested that using cycling, as a rehabilitation tool, could significantly improve lower body extremity function of stroke patients [8–12]. The pattern of cycling is very similar to walking [13–16] because both cycling and walking are cyclical and required reciprocal flexing and extension movements from the hip, the knee, and the ankle. Moreover, these exercises could also alternatively activate agonist and antagonist muscles with regular intervals of activity and coordination [17–19], which mitigated the balancing problem and provided a safer means for rehabilitation. Cycling exercise has great potential in preambulation training methods, commencing as soon as the patient has the ability to sit.

Though cycling exercise can potentially restore muscle strength, some problems still remain with this rehabilitation method. Cycling requires participants to move both of their lower limbs alternately with equal force, but, for hemiparesis patients, the lack of activity of the affected limb is often compensated by the unaffected limb. The unaffected limb may mask the insufficiency of the affected limb and result in uncoordinated training, which may reduce potential benefits and intensify gait dissymmetry of the hemiparesis patient [20, 21]. To attempt to solve this problem, a real-time feedback mechanism that could provide information concerning the cycling process would be helpful, which would remind the patients to focus on the task.

Virtual Reality (VR) provides patients with a more realistic, varied, and enhanced sensory perception experience and also facilitates motor learning based on various feedback mechanisms [22, 23]. It can simulate body movements of daily life, making rehabilitation more entertaining. VR training may also improve cortical reorganization and neuroplasticity by encouraging movement [24–26]. Related studies have proposed several advantages concerning the combination of VR and existing rehabilitation methods [27, 28]. It has been shown that combining VR with treadmill use, or other mechanical assistance, can increase stroke patients' walking speed and distance on even ground [29, 30].

VR-based treadmill ambulatory training provides suspension to support participant's trunk. However, the training sessions are often time-consuming and cumbersome, which not only make participants feel uneasy but also reduce their motivation. A study proposed a novel design to combine VR technology with bicycles, which effectively improves the cardiopulmonary function, the muscle strength, and the operational performance [31]. However, the participants in those studies were normal participants.

The cycling combining VR system has shown positive improvement for stroke patients. A recent study shows that when visual feedback is provided in cycling exercise, it will bring better cycling smoothness, and the average power output is also greater than the exercise without visual feedback [32]. For smoother cycling, bilateral leg force output balance can directly indicate cycling performance and is easy for patients to understand [33]. The force output status can be indicated with visual feedback during cycling training, which helps to improve pedaling balance and walking ability. However, some VR-cycling systems only show force output. Although the display can guide users to achieve training effects, the entertaining effects and gaming designs still require further improvement [32, 34]. Therefore, combining VR with a proper feedback device can provide a safer and more interesting training method for the stroke patients, which, in turn, allows the users to learn proper postures and functional abilities [35–39].

Therefore, the purpose of this study is to evaluate the training effect of customizable VR-Cycling Training System (VRCTS) for use in the rehabilitation of stroke patients, by comparing the result of the Asymmetry Ratio Index (ARI) of bilateral pedal force and force plate, before and after training. This system should allow clinical therapists to quantitatively measure the difference of force between both legs during cycling exercise, helping stroke patients to train the affected leg and track their progress. The interactive VR rehabilitation program gives feedback information to patients, stating their balance and speed condition for further customization. The interactive VR rehabilitation program might also offer interesting visual feedback to help patients focus on the training.

## 2. Experimental Section

### 2.1. Integration of the VR-Cycling Training System

**Figure 1:**
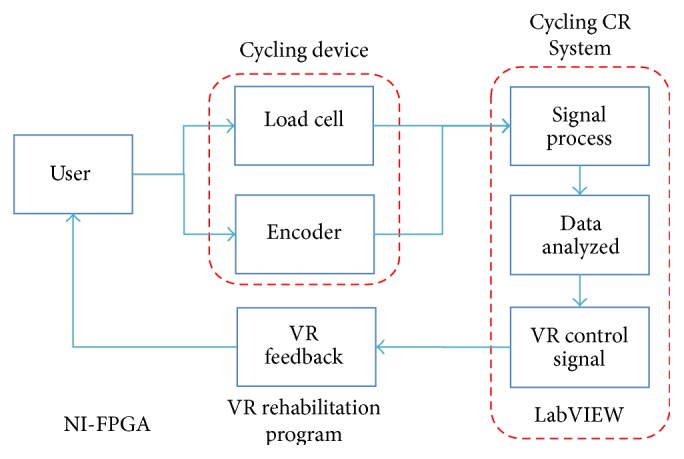
VRCTS system blocks.

**Figure 2:**
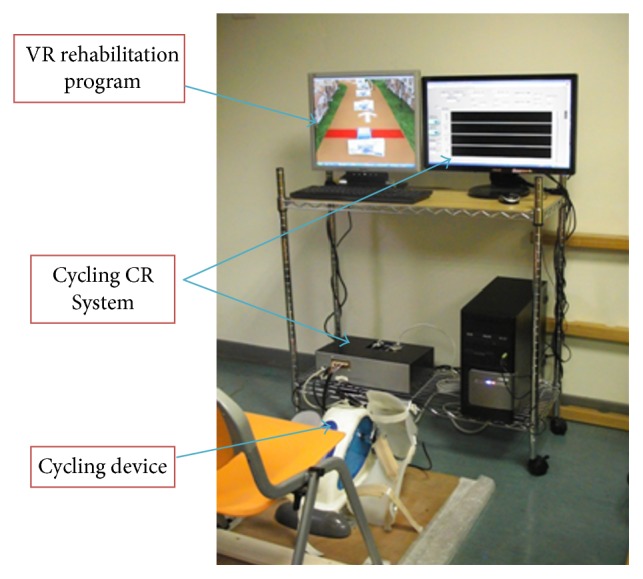
VRCTS system experiment setup, which includes VR rehabilitation program, Cycling CR System, and a cycling device.

The entire VR-Cycling Training System (VRCTS) consists of three components, as shown in Figures 1 and 2. The first component is a cycling device with sensors. The second component is a Cycling Graph User Interface Control and Data Record System (Cycling CR System), which serves as a data measuring and control module. The final component is a VR rehabilitation program that can provide real-time visual feedback.

### 2.2. Cycling Device

**Figure 3:**
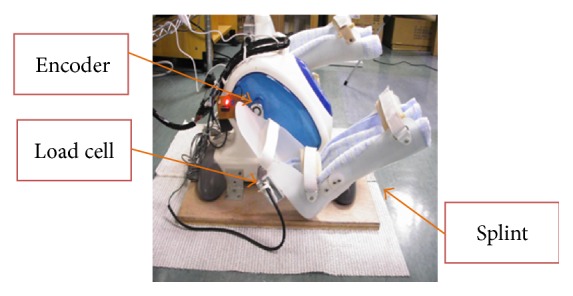
The cycling device which includes an encoder inside the cycling module, a load cell placed in both pedals, and a splint on top of each pedal.

The cycling device is shown in Figure 3. It is composed of three parts: a cycling device, two load cells placed inside each pedal to detect the force from user's feet, and an angle encoder attached to the crank of the cycling device.

To determine the stepping force, two load cells (MLP-100; Transducer Techniques, Inc., USA) were installed inside each pedal of the cycling device, and a splint was placed on the top of each pedal. When patients perform the cycling exercise, the output of cycling force can be accurately measured by using an amplifier and an adjustable circuit.

**Figure 4:**
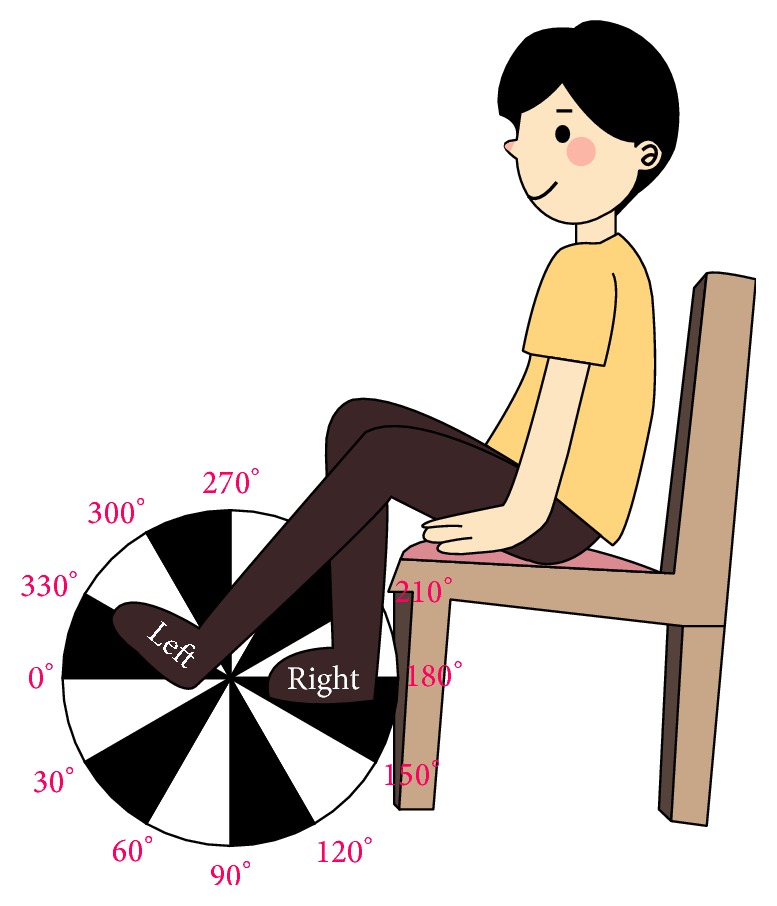
The relationship between the angle and the cycling position; left leg parallel to the ground is the 0 degree.

An encoder (MES 30-p; Microtech Laboratory Inc., Kanagawa, Japan) has three output signals which are phases A, B, and Z; it was applied to determine the cycling speed. The encoder was located in the middle of the cycling device and connected to the crank arm. Phases A and B are used for counting the angle and determined the direction. When the crank has been rotated 1 degree, phase A and phase B output signal will be turned from low voltage to high voltage. The relationship between the angle and the cycling position is shown in Figure 4. If a user moved the pedals clockwise, phase A would be half a duty cycle ahead of phase B. Otherwise, phase B would be a half duty cycle ahead of phase A. In a full pedal circle, the encoder counted 360 times; each count represented 1 degree; accordingly, we could determine the angle of the pedals. The 0-degree position is shown in Figure 4. When the encoder counted 360 times, the angle would be back to 0 degree, and phase Z turned its state to low voltage simultaneously. Phase Z was used to count the pedal cycle number. The cycling speed was calculated by Cycling CR System. If the speed was too low, it was considered as 0 RPM.

### 2.3. Cycling Graph User Interface Control and Data Record System

A Cycling Graph User Interface Control and Data Record System (Cycling CR System) was developed based on NI-FPGA system (National Instruments, TX, USA; compact RIO 9014) and LabVIEW software, which is developed for the clinician to set up the parameters for the Virtual Reality rehabilitation program and to follow the state of the participants. The Cycling CR System analyzed signals from the encoder and the load cell at a sample rate of 1 k Hz. The encoder signal was programmed to calculate cycling speed in rotations per minute (RPM). The cycling speed formula is shown below. A time parameter *t*_0_ is the start counting of each rotation at the point of 0 degree. A time parameter *t*_1_ is the end of each rotation at the point of 359 degrees. Consider

(1) $$\begin{array}{c} \text{Cycling speed}=\frac{60}{t_{1}-t_{0}}. \end{array}$$

**Figure 5:**
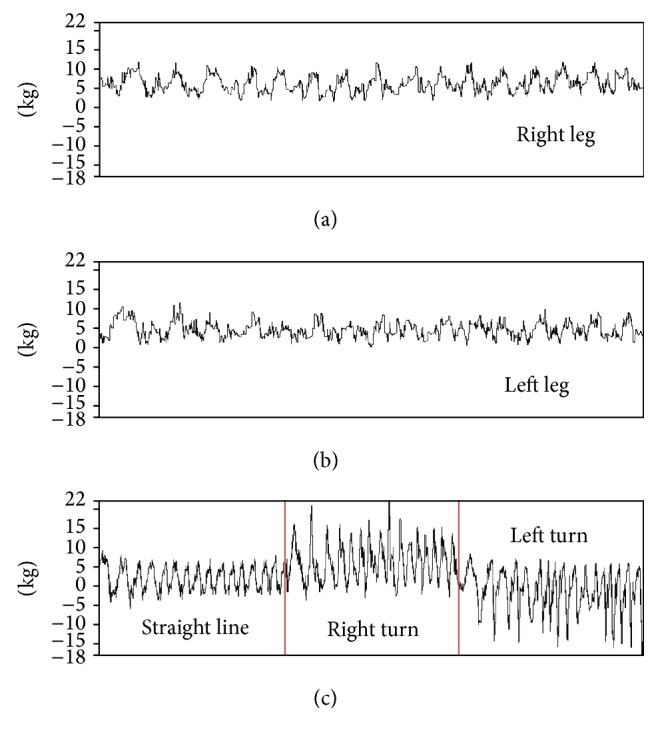
Cycling force value. (a) Right-leg force. (b) Left-leg force. (c) DF values. The first part is the DF of a straight line mode, the second part is DF in a right-curve mode, and the third part is DF in a left-curve mode.

For the load cell, we design the Cycling CR System to calculate the force in each leg and the Difference Force (DF) between the two legs. The DF formula is shown as follows:

(2) $$\begin{array}{c} \text{DF}=\frac{1}{N}\sum \left(\;{\text{R}}_{\text{force}}-{\text{L}}_{\text{force}}\;\right), \end{array}$$

where R_force_ is the right-leg cycling force (Figure 5(a)), L_force_ is the left-leg cycling force (Figure 5(b)), and *N* is the number of points in 0.1 seconds. When DF is a positive number, the right-leg cycling force is stronger than the left-leg cycling force. When DF is a negative number, the turning point is the threshold value of the left turn, which is one SD minus the negative left-leg force. The RTP (right turning point) is the threshold value of the right turn, defined by one standard deviation (SD) added to the average positive DF. The LTP (left turning point) is the threshold value of the left turn, defined by one standard deviation (SD) minus the average positive DF. Both the RTP and the LTP are calculated in the pretest. When the participants try to turn the VR car in VR rehabilitation program during turns, they have to generate greater strength with left leg or right leg. Therefore, the DF value during right turn or left turn will be calculated, and the right-turn DF or left-turn DF value is produced. When the right-turn DF is greater than the RTP, or if the left-turn DF is smaller than the LTP, a right or a left turning signal will be generated and transmitted to VR rehabilitation program and the VR car will turn 15 degrees. All the control signals will be passing from the Cycling CR System to the VR rehabilitation program.

### 2.4. Virtual Reality (VR) Rehabilitation Program

**Figure 6:**
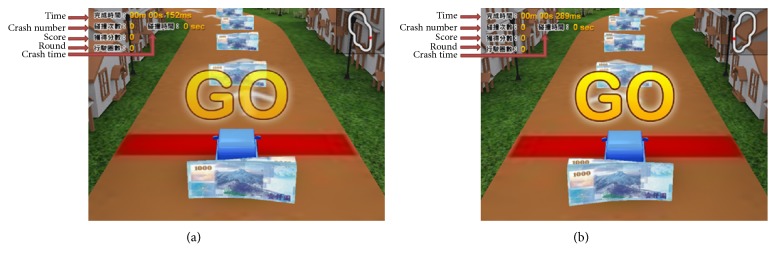
VR rehabilitation program courses. (a) Left-curve course. (b) Right-curve course.

A Virtual Reality (VR) rehabilitation program was applied in this system, which was used to provide participants with visual feedback. The VR rehabilitation program was created by the Virtools 4.0 (Dassault Systemes; Vélizy-Villacoublay, France). The VR display uses houses on both sides of the road in the program to give it an environment and NT$1000 New Taiwan dollar bills is set in the middle of the road as a target. A VR car was placed in the middle of the screen. We provided two courses, a right-curve course and a left-curve course, to collect notes, thus creating more opportunities for participants to train their affected leg, as shown in Figure 6. At the top of the VR rehabilitation program there are shown the game time, crash number, crash time, score, and round to give participants the motivation to improve with each attempt in the training process. The game time will show how long the participant has been training, but after it has been 15 minutes, the program will stop. Crash number shows how many times that car has hit a house. Crash time shows how often, in seconds, that the VR car has hit a house. When gathering the dollar bills produces a score of 5 points, it is an indication of the user's ability to control the car's position, since the participant has had to keep the VR car in the middle of the road, whether on straight or curvy road by learning how to turn the car. This forces them to train the weak side of leg thus satisfying the requirements of their rehabilitation.

**Figure 7:**
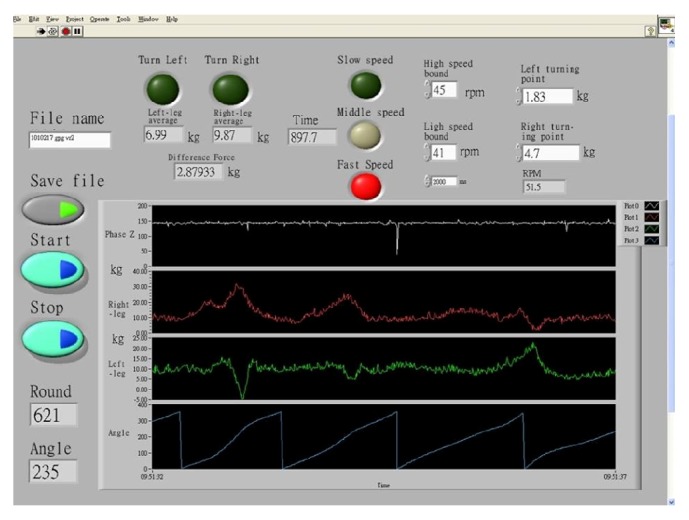
Cycling CR System; since it is for the clinician to set up the GUI, this shows the cycling force output and speed and how to set up the parameters for the VR rehabilitation.

**Figure 8:**
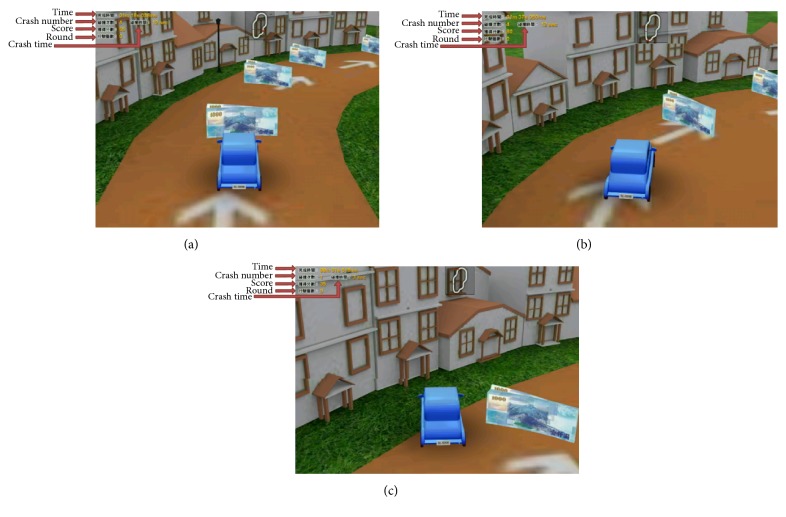
Stroke patients control the VR car to pass a curve. (a) Before turning. (b) After turning. (c) Stuck in the curve.

The Cycling CR System was also used to control the VR car. A few parameters were applied to the setup in the Graph User Interface (GUI), as shown in Figure 7. The parameters were used for right-turn control, left-turn control, and speed control. When the Cycling CR System sent the turning signal to the VR rehabilitation program for a turning control, the VR car turned 15 degrees. In Figure 8(a) showing VR car in a right curve, if the right-turn DF is greater than the RTP, VR car will turn 15 degrees (Figure 8(b)) to make the VR car pass the curve, or the VR car will become stuck in the curve (Figure 8(c)).

There were three levels for speed control, and high speed bound and low speed bound were set by the pretest parameters. If the cycling speed was within the high speed bound and the low speed bound, the VR car would move at speed level 2 (middle speed). If cycling speed was faster than the high speed bound, the VR car would move according to speed level 1 (fast speed). On the other hand, if cycling speed was slower than the low speed bound, the VR car would move at speed level 3 (slow speed). Though if cycling speed is slower than 15 RPM, the VR car stops. All cycling signals are processed and controlled by the Cycling CR System.

### 2.5. Evaluation Task and Statistics

This study adopted quasiexperimental, pretest, posttest nonequivalent control group design. Use the nonblind method to compare the control group and experiment group. Participants were tested before and after the intervention and in a follow-up assessment one week after the end of the treatment by means of the following assessment tests:

(1) A bilateral pedaling test was done, where patients will cycle for 2 rounds of 17 cycles. The first round is to let the patient get used to the pedaling exercise. The 5~10 cycles in 2nd round will be used to record the data of the affected and unaffected leg pedaling force ARI (Asymmetry Ratio Index):
  (3) $$\begin{array}{c} \text{ARI}=\left|\;1-\frac{\text{affected side}}{\text{unaffected side}}\;\right|. \end{array}$$
(2) To measure the stand balance ARI, the force plate (zebris force measure platform, zebris Medical GmbH, Germany) and zebris WinFDMS (zebris Medical GmbH) were used. The sample rate is 1000 Hz. The patient stands and distributes his weight across a force plate, once in place, stands for thirty seconds to adjust their position, then closes their eyes, and remains still for ten seconds to record the data showing the average COP length and COP area for both legs.

To analyze the result of pedal force and force plate, the SPSS 14.0 (SPSS Inc., Chicago) was used, to analyze the data between before and after training, with the pair *t*-test statistics method to compare the results, if *p* < 0.05 means that the result is significant difference.

### 2.6. Participants

The feasibility of VR-Cycling Training System (VRCTS) was tested by ten stroke patients, who were separated into two groups: the first group is the control group and the second group is the experimental group.  Table 1 shows the characteristics of participants in the experiment.

In the control group, there were three participants; the average age was 61.3 ± 6.1, ranging from 56 to 68. This group consisted of two females and 1 male. The average month after stroke was 13.3 ± 4.16. The functional ability of the participants' lower limb was classified by Brunnstrom stage classification. Three participants were in stages III and IV on the Brunnstrom stage classification.

In the experimental group, there were six participants, two participants with right side affected and four participants with left side affected. The average age of this group was 54 ± 9.14, and this group consisted of five females and one male. The average month after stroke was 15 ± 10.6. The functional ability of the participants' lower limb was in stages III and IV on the Brunnstrom stage classification.

### 2.7. Design and Procedure

Stroke patients were recruited from the Chung Shan Medical University Hospital to participate in this study. A diagnosis of stroke with Brunnstrom stage III of the lower extremity and no significant perceptual, cognition, or sensory problem was selected. The Institutional Review Board for Human Studies at Chung Shan Medical University Hospital has approved this protocol (number CS11034), and all the participants and their caregivers provided informed consent.

The control group and experimental group both had the same rehabilitation treatments five times per week, with time being for 1 hour. The experimental group also had VRCTS training besides their rehabilitation treatment three times a week for 15 minutes each time for a total of ten times. For VRCTS training, a pretest will be performed to help adjust the parameter in the VRCTS system.

In the pretest, all participants were asked to perform 15 cycles. The initial 0~4th cycles helped the participants to turn the pedals smoothly and got accustomed to the exercise. The 5th~10th cycles were recorded and used to measure and calculate the parameters. The 11th~15th cycles were to prevent cycling speed deceleration during the recorded period. When the cycling angle was 0~30 degrees, the average force output of the left leg was extracted. When the cycling angle reached 180~210 degrees, the average force output of the right leg was extracted. All of the above values were measured by the Cycling CR System to distinguish the difference of the force output between two legs. Then, the DF was computed for the average cycling speed (RPM) and for the turning threshold values, which are the RTP and the LTP. The parameter range of high and low speed was also calculated in the pretest. Then, these participants were asked to look at the VR rehabilitation program and control the VR car with the cycling device for 15 minutes.

## 3. Results

### 3.1. DF in VR Rehabilitation Program

The LTP and the RTP were determined in the pretest. During the VR rehabilitation program, the DF of the right and the left turns would be evaluated, and the results are shown in Table 2.

In the pretest, the cycling in a straight line, average DF value in left affected side and right affected side is 2.96 ± 0.31 kg and 0.49 ± 0.08 kg. The average RTP value in left affected side and right affected side is 3.82 ± 0.32 kg (the unaffected side) and 1.34 ± 0.75 kg (the affected side), and the average LTP value was −0.79 ± 0.27 kg (the affected side) and −2.77 ± 0.48 kg (the unaffected side). In a straight line condition, users could go straight by controlling the DF between RTP and LTP. On the other hand, during the turning moment, users could control the DF larger than the right or left turning point. Therefore, with this technique, users were able to control the VR car.

In the VR rehabilitation program, during the right turning moment the average right-turn DF for the left and right affected side were 6.43 ± 2 kg (the unaffected side) and 1.89 ± 0.83 kg (the affected side). In the left turning moment, the average left-turn DF were −2.19 ± 0.46 kg and −5.07 ± 0.07 kg, respectively. And the total average cycling speed was 63.25 ± 2.36 RPM and 53 ± 8.48 RPM; if the patient can cycle faster and control the VR car's direction with skills, then patients can finish more rounds, and more scores. Also with the different speeds, the VR car can provide patients with motivation to cycle faster and have better clinical effects.

### 3.2. Evaluation

The stroke patient bilateral pedal force results from before and after the ten VRCTS training sessions are shown in Table 3, the ARI in average results of the symmetry before training between legs in control group and experimental group are 0.24 ± 0.22 and 0.34 ± 0.2, and while the results after training are 0.22 ± 0.20 and 0.12 ± 0.07, in the control group, the symmetry is 0.02 (*p* = 0.5) which did not improve and did not significantly differ in results. In the experimental group, symmetry improved by 0.22 (*p* = 0.031) which was significantly different where the control group results were closer to 0. This showed that the pedal force symmetry had improved after training with the VRCTS.

The results of percentage parameter detected by the force plate are shown in Table 4. The force plate average ARI results after training are 0.23 ± 0.01 and 0.05 ± 0.03. In the control group, the ARI in force plate increased by 0.01 (*p* = 0.73) so it did not differ significantly. In experimental group, the ARI result had improved by 0.29 (*p* = 0.046) so there is a significant statistical difference, which showed that, after the VRCTS training, the standing balance ability has increased.

Descriptive statistics revealed that the changes in the bilateral pedal force coordination and force plate after the VRCTS training were comparable in both groups. The bilateral pedal force and force plate results after training increased in the experimental group while those in the control group remained almost the same.

## 4. Discussion

In this study, we presented a VR-Cycling Training System (VRCTS), which consisted of a VR rehabilitation program and the cycling. In this system, the cycling was used to control a VR car and to give users feedback. To verify the system functionality, 10 stroke patients participated in this experiment. The results showed that, after the calibration of the pretest, users could control the direction and the speed of the VR car, which demonstrated that the system might work on stroke patients.

Many studies have reported that immediate rehabilitation training after the onset of stroke can help patients to restore functional ability faster. Cycling is similar to gait [8–10], while it only requires performers to be able to sit. Therefore, the cycling rehabilitation training of lower limb may be an effective way to improve muscle strength and balancing ability. However, there are still some problems in cycling exercise. For example, sometimes stroke patients will use the unaffected side to compensate the affected side [8].

To remedy the flaws of other cycling systems for lower limb, we designed a cycling device equipped with load cells and encoder to detect the cycling force and speed of users in real-time. In the force output analysis of lower limbs, a pretest was used for calibration, so the force output of users could be successfully distinguished in the following tests. DF values could determine if users used their unaffected limb to compensate the affected one. Thus, the problems of cycling for lower limb could be solved by analyzing and evaluating the detected force output from both legs, and users would receive feedback to remind them of using the affected leg.

This study combines cycling with VR to help users to enhance concentration and motivation during rehabilitation training. One common problem of VR is that the display is often plain and unattractive [22]. In the previous study [34], a VR feedback display was used to remind user of their cycling force balance. In the VR display of the system, the force output was displayed as two bars. Although this display could guide users to achieve training effects, its entertainment effect and gaming design still require further improvement. Moreover, one system [31] used a bicycle and several sensors with a VR game. Although the game was quite entertaining, the system applied a conventional bicycle for the training. The seat of the bicycle was relatively small, which might not be able to provide the stability required by stroke patients. In addition, the height of the seat was too high, which made it difficult for patients to mount and dismount. VRCTS used Virtools to design a VR rehabilitation program, and cycling could be used to control the VR car for training, which could enhance the entertainment effect and motive of patient for training. The cycling device could provide a convenient and safe environment with any kind of chair. Even a wheelchair could be used with this cycling device for patients who had sitting balance problems. The VR rehabilitation program also scheduled more turning for the affected side of patients, so they could train their muscle strength and balance of the affected side.

In many existing cycling systems, the normal leg often compensates for the affected one in cycling training. Moreover, the system parameter setting of the VR rehabilitation program is also difficult to operate. The VRCTS developed in this study uses pretest for calibration, so the system can be customized according to the need of each individual patient. The data obtained through the 15 pedal cycles performed in the pretest was analyzed to attain the right-turn and left-turn settings, so cycling alone is used to control the speed and the direction of VR car in the program.

During the 15 minutes of the training process, the Cycling CR System can give the VR rehabilitation program a signal to determine the speed and the direction of the VR car. The participants will have the feedback on the direction and the speed of the VR car and direction of the car will let the participant know which leg should increase cycling force to control the desired direction to gather the $1000 New Taiwan dollar bills and successfully turn curve; then VR rehabilitation program will show the information of game time, crash number, crash time, score, and round, and this information will show the performance of the program.

However, the parameter setting is determined by individual pretest. In the left side and right side affected, VR program average right-turn DF are 6.43 ± 2 kg and 1.89 ± 0.83 kg, and the RTP are 3.82 ± 0.32 kg and 1.34 ± 0.75 kg. The average left-turn DF is −2.19 ± 0.46 kg and −5.07 ± 0.07 kg and the LTP is −0.79 ± 0.27 kg and −2.77 ± 0.48 kg. However, the result shows that, among the six patients, the strength of the unaffected leg is stronger than the affected one, but this study shows that stroke patients can perform the controlling function; thus during turning curve the DF in the affected side can still be greater than the unaffected side. Since the game is training-oriented, this system might be challenging for hemiparesis patients because the force output of the affected side needs to be greater than the turning point, so the system can provide therapeutic effects.

In 2011, 153 patients with chronic stroke participated in [34] and they performed the visual feedback cycling training for 14 minutes twice a week. The patients should continue the training for two weeks, which were six times in total. The evaluation result showed that the method of the pedal torque symmetry was to maintain the rotating speed at 30 RPM for two minutes, and the torque would be calculated separately. After the training, the patients were divided into three groups, and one participant in each group was selected to conduct discussion. After the treatment, the pedal force was more symmetrical than that of the pretest and showed significant difference (*p* < 0.01) [34]. The result of the experimental group in our study also can support the hypothesis of the study that the adoption of visual feedback could improve the cycling performance.

The participants of the experimental group in this study received the VRCTS treatment. Their average value of the pedal force of the affected side increased from 11.85 ± 3.92 kg to 15.09 ± 1.86 kg, and the Asymmetry Ratio Index (ARI) improved from 0.34 to 0.12, which showed significant difference (*p* = 0.031). Regarding the participants of the control group, their average value of the pedal force of the affected side increased from 9.27 ± 1.88 kg to 9.42 ± 2 kg and did not show significant difference (*p* = 0.59). From the results of previous studies, we can infer that the reason of the improvement of the test group is that after the vision goes into the brain, the participants will practice repeatedly to prevent the collision of edge of marching objects in Virtual Reality. In addition, the visual reinforcement feedback of receiving virtual NT$1000 New Taiwan dollar bills by maintaining the VR car in the middle of the road activates the premotor cortex of the brain, which provides for real-time action reaction. Therefore, the coordination between lower limbs increases, and the visual reinforcement feedback can stimulate human brain remodeling, which makes the brain maintain good symmetry without visual feedback.

In the force plate, if the plantar pressure distribution approaches 50% means the symmetry is the best. Regarding the participants of the test group in this study, after the treatment, the plantar pressure distribution of the affected side improved from 40.75 ± 10.51% to 48.73 ± 0.79%, and Asymmetry Ratio Index (ARI) showed significant difference (*p* = 0.046). However, regarding the participants of the control group in this study, the plantar pressure distribution of the affected side improved from 43.2 ± 1.33% to 44.1 ± 0.49% and did not show significant difference (*p* = 0.73).

In both evaluations proved, this system has been shown to improve the problem of leg compensation and achieve customized training, which helps stroke patients to operate it. The VRCTS also makes the cycling training more entertaining and helps users to concentrate more on the rehabilitation training.

## 5. Conclusions

The developed Virtual Reality-Cycling Training System in this study can improve the symmetry of the bilateral pedal force from the cycling detection significantly, and the performance is better than the control group. In addition, the distribution ARI of bilateral pedal force and force plate is improved significantly. The result shows that the treatment of the Virtual Reality-Cycling Training System can increase the bearing symmetry in static balance effectively, which provides a new choice for future clinical rehabilitation treatment. However, since the study adopted convenience sampling, nonblind design, and nonrandomized grouping for the participants, the deviation in the results caused by the interaction between participants cannot be excluded totally. Moreover, the maturation effect of personal recovery status and the number of samples of the two groups recruited in this study are not equal. The number of samples of the control group is insufficient, so we cannot be sure that the result complies with normal distribution.

## Figures and Tables

**Table 1:** Participants' data.

Subject	Age (years)	Gender	Affected side	Month after stroke	Brunnstrom stage	Height (cm)	Weight (kg)
Control group							
1	60	F	R	12	3	152	75
2	56	M	R	18	4	167	80
3	68	F	R	10	4	156	69
Mean (SD)	61.3 (6.1)			13.3 (4.16)		158.4 (7.76)	74.6 (5.5)

Experimental group							
1	58	F	L	12	4	154	80
2	42	F	L	6	4	158	76
3	51	F	L	12	4	154	70
4	62	F	L	12	3	155	72
5	65	F	R	36	4	156	65
6	46	M	R	12	3	163	82
Mean (SD)	54 (9.14)			15 (10.6)		156.67 (3.4)	74.17 (6.4)

**Table 2:** Evaluation of the VR rehabilitation program.

Stroke	Affected side	Straight line DF (kg)	Right/left turning point DF (kg)		Right-turn/left-turn DF (kg)		Cycling speed (RPM)
			Left	Right	Left	Right	
1	L	2.6	−0.77	4.02	−2.85	5.14	63
2	L	2.8	−0.65	3.6	−1.8	9.34	65
3	L	3.26	−0.59	4.17	−2.08	6.15	65
4	L	3.18	−1.18	3.5	−2.03	5.07	60
Mean (SD)		2.96 (0.31)	−0.79 (0.27)	3.82 (0.32)	−2.19 (0.46)	6.43 (2)	63.25 (2.36)

5	R	0.43	−3.11	0.81	−5.12	1.3	47
6	R	0.55	−2.43	1.87	−5.02	2.48	59
Mean (SD)		0.49 (0.08)	−2.77 (0.48)	1.34 (0.75)	−5.07 (0.07)	1.89 (0.83)	53 (8.48)

**Table 3:** Bilateral pedal force results.

Group	Affected side (kg)	Unaffected side (kg)	ARI	Affected side (kg)	Unaffected side (kg)	ARI	*p* value
	Pretest	Pretest		Posttest	Posttest		
Control group							
Average	9.27	12.22	0.24	9.42	12.34	0.22	0.59
SD	1.88	2.23	0.22	2.89	1.53	0.20	
Experiment group							
Average	11.85	18.18	0.34	15.09	16.98	0.12	0.031
SD	3.92	1.71	0.2	1.86	0.76	0.07	

**Table 4:** Force plate results.

Group	Affected side (%)	Unaffected side (%)	ARI	Affected side (%)	Unaffected side (%)	ARI	*p* value
	Pretest	Pretest		Posttest	Posttest		
Control group							
Average	43.2	56.8	0.24	44.1	55.9	0.23	0.73
SD	1.33	1.33	0.04	0.49	0.49	0.01	
Experiment group							
Average	40.75	59.25	0.34	48.73	51.27	0.05	0.046
SD	10.51	10.51	0.22	0.79	0.79	0.03	
